# Ad-VT enhances the sensitivity of chemotherapy-resistant lung adenocarcinoma cells to gemcitabine and paclitaxel in vitro and in vivo

**DOI:** 10.1007/s10637-021-01204-4

**Published:** 2022-01-04

**Authors:** Gaojie Song, Chao Shang, Lili Sun, Yiquan Li, Yilong Zhu, Zhiru Xiu, Zirui Liu, Yaru Li, Xia Yang, Chenchen Ge, Jinbo Fang, Ningyi Jin, Xiao Li

**Affiliations:** 1 Medical College, Yanbian University, Yanji, China; 2 Changchun Veterinary Research Institute, Chinese Academy of Agricultural Sciences, Changchun, China; 3 Academician Workstation of Jilin Province, Changchun University of Chinese Medicine, Changchun, China; 4 Department of Head and Neck Surgery, Tumor Hospital of Jilin Province, Changchun, China

**Keywords:** Lung cancer cells, Oncolytic virus, Apoptosis, Autophagy, P-gp

## Abstract

*Background* One of the main challenges in the clinical treatment of lung cancer is resistance to chemotherapeutic drugs. P-glycoprotein (P-gp)-mediated drug resistance is the main obstacle to successfully implementing microtubule-targeted tumor chemotherapy. *Purpose* In this study, we explored the effect of Ad-hTERTp-E1a-Apoptin (Ad-VT) on drug-resistant cell lines and the molecular mechanism by which Ad-VT combined with chemotherapy affects drug-resistant cells and parental cells. *Methods* In vitro, cell proliferation, colony formation, resistance index (RI), apoptosis and autophagy assays were performed. Protein expression was analyzed by Western blotting. Finally, a xenograft tumor model in nude mice was used to detect tumor growth and evaluate histological characteristics. *Results* Our results showed that Ad-VT had an obvious killing effect on A549, A549/GEM and A549/Paclitaxel cancer cells, and the sensitivity of drug-resistant cell lines to Ad-VT was significantly higher than that of parental A549 cells. Compared with A549 cells, A549/GEM and A549/Paclitaxel cells had higher autophagy levels and higher viral replication ability. Ad-VT decreased the levels of p-PI3k, p-Akt and p-mTOR and the expression of P-gp. In vivo, Ad-VT combined with chemotherapy can effectively inhibit the growth of chemotherapy-resistant tumors and prolong the survival of mice. *Conclusions* Thus, the combination of Ad-VT and chemotherapeutic drugs will be a promising strategy to overcome chemoresistance.

## Introduction

Lung cancer is one of the most common malignant tumors the worldwide. Non-small cell lung cancer (NSCLC) accounts for approximately 80% of all lung cancers and causes 1.7 million deaths worldwide annually. According to the World Health Organization (WHO), the number of new cases in 2020 was 2,206,771 (11.4%), the number of deaths was 1796144 (18%). Lung cancer is the leading cause of cancer-related death primarily due to drug resistance, which has always been a major obstacle to successful cancer treatment. The vast majority of patients will gradually develop drug resistance to chemotherapy or targeted drugs even if they show a good initial response [1]. Multidrug efflux is one of the most important mechanisms of chemotherapy resistance and functions by exporting chemotherapeutic drugs from cells, thus protecting them from their antitumor activities [2, 3]. P-glycoprotein (P-gp) is a typical multidrug resistance (MDR) protein that can reduce the intracellular concentration of chemotherapy drugs. Therefore, many scientists are investigating ways to alter P-gp function and expression and reverse chemotherapy resistance.

Oncolytic viruses (OVs), which selectively kill tumor cells without exerting any toxic effects on normal cells or tissues, are emerging as important agents in cancer treatment and have shown encouraging safety profiles in clinical trials [4–7]. In previous studies, our team designed and constructed the oncolytic adenovirus Ad-hTERTp-E1a-Apoptin (Ad-VT). Ad-VT contains an E1a gene controlled by the human telomerase reverse transcriptase promoter, which is highly active in more than 85% of human cancers but not in most normal human cells. In addition, Ad-VT expresses apoptin, a 13.6 kDa protein that is the product of the VP3 gene of chicken anemia virus (CAV) and has the ability to selectively kill a variety of human tumors or transformed cells but little cytotoxic effect on normal cells [8]. Therefore, Ad-VT is a new type of dual-specific oncolytic adenovirus with tumor-specific targeting and killing functions. As an adjuvant and alternative drug, Ad-VT has been studied for its antitumor effect; the results indicate that Ad-VT can inhibit the proliferation of malignant tumor cells in vitro and in vivo. Ad-VT mitigates the growth of tumor cells through a variety of mechanisms, including inducing apoptosis and autophagy, inhibiting the proliferation and differentiation of tumor cells, inducing cell cycle arrest, preventing angiogenesis, and hindering cell energy metabolism. However, the mechanism by which Ad-VT affects drug-resistant lung cancer has not been reported.

This study aims to (1) detect P-gp expression in NSCLC tissues and cell lines and study its biological effects through a series of in vitro and in vivo experiments, (2) evaluate the cytotoxicity of Ad-VT on lung cancer cells in vitro, (3) investigate the mechanism of Ad-VT in chemotherapy-resistant lung cancer cells, and (4) determine the therapeutic effect of Ad-VT combined with gemcitabine or paclitaxel in an in vivo lung tumor model.

## Materials and methods

### Cells, viruses and animals

A549 cell lines were purchased from the Cell Bank of the Shanghai Institute for Biological Sciences. A549/GEM and A549/Paclitaxel cell lines were purchased from the BeNa Culture Collection. The recombinant adenovirus Ad-VT (Ad-hTERTp-E1a-Apoptin) was constructed and preserved in our laboratory (Changchun Veterinary Research Institute, Chinese Academy of Agricultural Sciences, Changchun, China) [9]. Female BALB/c nude mice aged 4 to 5 weeks were purchased from the Experimental Animal Center of the Academy of Chinese Academy of Agricultural Sciences.

### Drugs, reagents and antibodies

Gemcitabine and paclitaxel were purchased from MedChemExpress LLC. Protease inhibitor cocktail and phosphatase inhibitor cocktail were purchased from APExBIO Technology LLC. Primary antibodies against p-mTOR, mTOR, p-AKT, AKT, p-PI3K, PI3K, MDR1, cleaved caspase-3, Beclin-1, LC3A/B and GAPDH were purchased from Cell Signaling Technology, Inc.

### Crystal violet staining

Cells were seeded in 12-well plates, treated with Ad-VT (MOI of 80) for 48 h, and then stained with 0.4% crystal violet for 10 min at room temperature. The dye solution was discarded, and the stained cells were washed with PBS, dried and imaged.

### Hoechst assay

Cells in the logarithmic growth phase were cultured in 12-well cell culture plates, and 300 µl of Hoechst staining fluid was added to each well and incubated for 15 min. The dye solution was removed, and the cells were air dried and imaged.

### Flow cytometric analysis

Induction of apoptosis was detected by flow cytometry using a FITC Annexin V Apoptosis Detection Kit I. Briefly, cells were seeded in 6-well plates and treated with Ad-VT (MOI of 80) for 24, 48, or 72 h. Before analysis, the cells were stained with 5 µl of propidium iodide (PI) solution and 5 µl of Annexin V-FITC. The samples were then examined by flow cytometry for apoptosis analysis. Each experiment was repeated at least three times.

### CCK-8 assay

The CCK-8 assay was performed as described [10]. Briefly, cells were seeded in 96-well plates and treated with Ad-VT or drugs (gemcitabine and paclitaxel). Then, 10 µl of CCK-8 solution was added to each well and the cells were incubated for 3 h. Viable cells were assessed using a microplate reader at an absorbance of 595 nm.

### Monodansylcadaverine (MDC) detection

Cells were seeded on microscope cover slips in 6-well plates and cultured for 24 h, after which the medium was discarded. Then, the cells were washed three times with 0.1 mol/l of cold PBS before autophagic vacuoles were labeled with 0.05 mmol/l MDC. The percentage of cells with MDC-labeled autophagic vacuoles among the total cell population was calculated at 400 × magnification. Cells with 20 or more MDC markers were considered to have positive fluorescence.

### Western blot analysis

Total protein extraction from cells and Western blot analysis were performed as previously described [11, 12]. Protein from cells was extracted using the Minute™ Total Protein Extraction Kit for Animal Cultured Cells and Tissues. Briefly, 30 μg of total protein was separated via SDS/PAGE though 12% gradient gels and electrotransferred to nitrocellulose membranes. The membranes were blocked with NcmBlot blocking buffer before they were incubated with the appropriate primary antibody at 4 °C overnight. Then, the membranes were incubated with HRP-conjugated secondary antibody for 40 min at room temperature and washed with TBST three times. We calculated the band intensities of the proteins using ImageJ software (National Institutes of Health, USA) and normalized them to the intensity of the GAPDH band.

### Measurement of viral titers

A549, A549/GEM and A549/Paclitaxel cells were seeded into 6-well plates at a density of 2 × 10^5^ cells per well (n = 3 per condition). After 18 h of incubation, the cells were infected with Ad-VT at 10 MOI. After 24, 48 or 72 h of infection, the cells and supernatant were collected and then frozen and thawed 3 times. HEK-293 cells were seeded into 96-well plates at a density of 5 × 10^3^ cells per well. After 18 h of incubation, the cells were infected with tenfold diluted Ad-VT. The virus titers were calculated with the Reed-Muench method and expressed as TCID_50_ per milliliter of supernatant. In the 3-MA-treatment groups, the cells were pretreated with 3-MA for 2 h before they were subjected to Ad-VT infection.

### Analysis of apoptosis by TUNEL staining

Cells were seeded on microscope cover slips in 6-well plates. After 24 h of culture, the medium was discarded, and the cells were fixed with immunostaining fixative for 30 min and washed three times with cold PBS. Then, a TUNEL assay was performed using colorimetric TUNEL apoptosis assay kits according to the manufacturer’s instructions. Green fluorescence indicates TUNEL-positive cells, and blue fluorescence indicates nuclei with DAPI staining. In addition, a TUNEL assay with tumor sections was performed as previously described [13]. Images were obtained under a fluorescence microscope, and at least three random fields from each section were examined at 200 × magnification.

### Xenograft model

A549, A549/GEM and A549/Paclitaxel cells (1 × 10^7^) were subcutaneously inoculated into the right flank of female BALB/c nude mice (4–5 weeks, 20 ± 2 g); 7 days later, these mice were randomly divided into different groups (n = 10 per group) and then subjected to the indicated treatments. The tumor volume was measured every 5 days with calipers and calculated with the following formula: [(W2 × L)/2; W, width; L, length; in cubic millimeters]. In the Ad-VT-treated groups, 1 × 10^9^ plaque-forming units of Ad-VT were intratumorally injected in a volume of 100 µl in PBS every 3 days for a total of 10 treatments. In the drug-treated groups, gemcitabine (10 mg/kg) and paclitaxel (5 mg/kg) in corn oil were intraperitoneally (i.p.) injected in a volume of 100 µl. The drugs were given every 3 days for a total of 10 treatments. Tumor tissues (n = 3) were harvested 30 days after the initial virus injection and fixed in 4% paraformaldehyde in neutral buffer for 24 h.

### Histopathological analysis

Histopathological analysis of the tissue sections was performed as previously described [14]. Briefly, fixed tumor tissues were embedded in paraffin and sliced to 5 µm thickness, after which the sections were blocked with normal horse serum for 1 h and then incubated with antibodies against Ki-67 or P-gp at 4 ℃ overnight. After washing, the sections were incubated with secondary antibodies for 1 h and then washed with cold PBS. The proteins were visualized by incubating sections with DAB for 15–20 min at room temperature followed by counterstaining with hematoxylin. Slices were observed under a light microscope to detect Ki-67 and P-gp expression.

### Statistical methods

All data are presented as the mean ± SEM unless otherwise indicated, as Pan et al. previously described [15]. Differences between two groups were evaluated for statistical significance using a two-tailed unpaired t-test. When the differences among three or more groups were evaluated, one-way ANOVA was used. Survival data were subjected to Kaplan–Meier survival analysis. A *p* < 0.05 indicated statistical significance.

## Result

### Gemcitabine and paclitaxel sensitivity

A549, A549/Paclitaxel and A549/GEM cell lines were treated with different concentrations of paclitaxel or gemcitabine. The results showed that the IC_50_ values of paclitaxel in A549, A549/GEM and A549/Paclitaxel cells were 31.441 ± 1.72, 20.22 ± 1.48 and 3193 ± 207.6 nm/ml, respectively, and the IC_50_ values of gemcitabine were 481.3 ± 88.35, 8369 ± 474.6 and 406.2 ± 87.11 nm/ml, respectively (Table 1). The resistance index (RI) of A549/GEM cells to gemcitabine and paclitaxel was 17 and 1, respectively, and that of A549/Paclitaxel cells to paclitaxel and gemcitabine was 103 and 1, respectively. This finding that A549/GEM cells are sensitive to paclitaxel and that A549/Paclitaxel cells are sensitive to gemcitabine.

### P-gp expression was higher in cancer and was associated with shorter overall survival

**Fig. 1:**
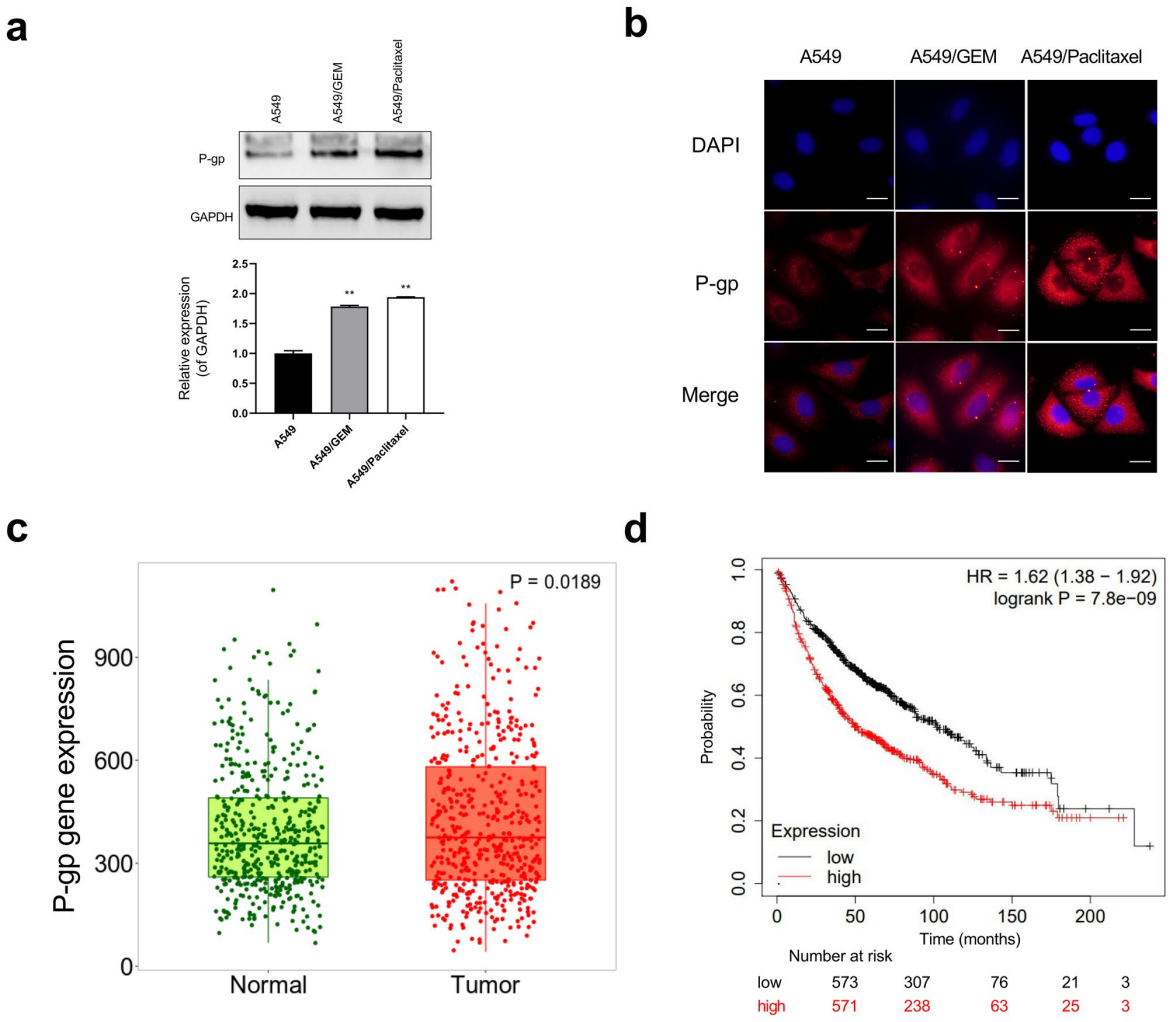
P-gp is overexpressed in A549/GEM and A549/Paclitaxel cells. **a** Western blot analysis of P-gp levels in A549, A549/GEM and A549/Paclitaxel cells. **b** Immunofluorescence analysis of P-gp expression (red fluorescence) and nuclei (blue fluorescence, DAPI staining) in A549, A549/GEM and A549/Paclitaxel cells. Scale bars: 25 µm. **c** P-gp expression in normal lung tissue and lung cancer specimens. Images were derived from The Human Protein Atlas (Kaplan–Meier Plotter) online database. **d** Kaplan–Meier survival curves and log rank tests were established to assess the correlation between P-gp expression and overall survival of lung cancer patients. Each experiment was repeated three times. **p < 0.01

P-gp, which reduces the intracellular concentrations of chemotherapeutic agents, is the classical MDR protein. Western blot results demonstrated that P-gp expression in A549/Paclitaxel and A549/GEM cells was significantly higher than that in A549 cells (Fig. 1a). Immunofluorescence staining was used to detect P-gp expression, as shown in Fig. 1b, and the results were consistent with the Western blot results. To determine the clinical relevance of P-gp, we analyzed its protein expression in clinical specimens, which was significantly higher in lung adenocarcinoma (LUAD) tissues than in normal lung tissues (Fig. 1c). Furthermore, to assess the clinical significance of P-gp expression in lung cancer, NSCLC patients were further divided into high P-gp expression and low P-gp expression groups based on the mean expression. As shown in Fig. 1d, Kaplan–Meier analysis revealed that high P-gp expression was significantly correlated with poor overall survival in patients with lung cancer.

### Ad-VT had a significant inhibitory effect on chemoresistant cell lines

**Fig. 2:**
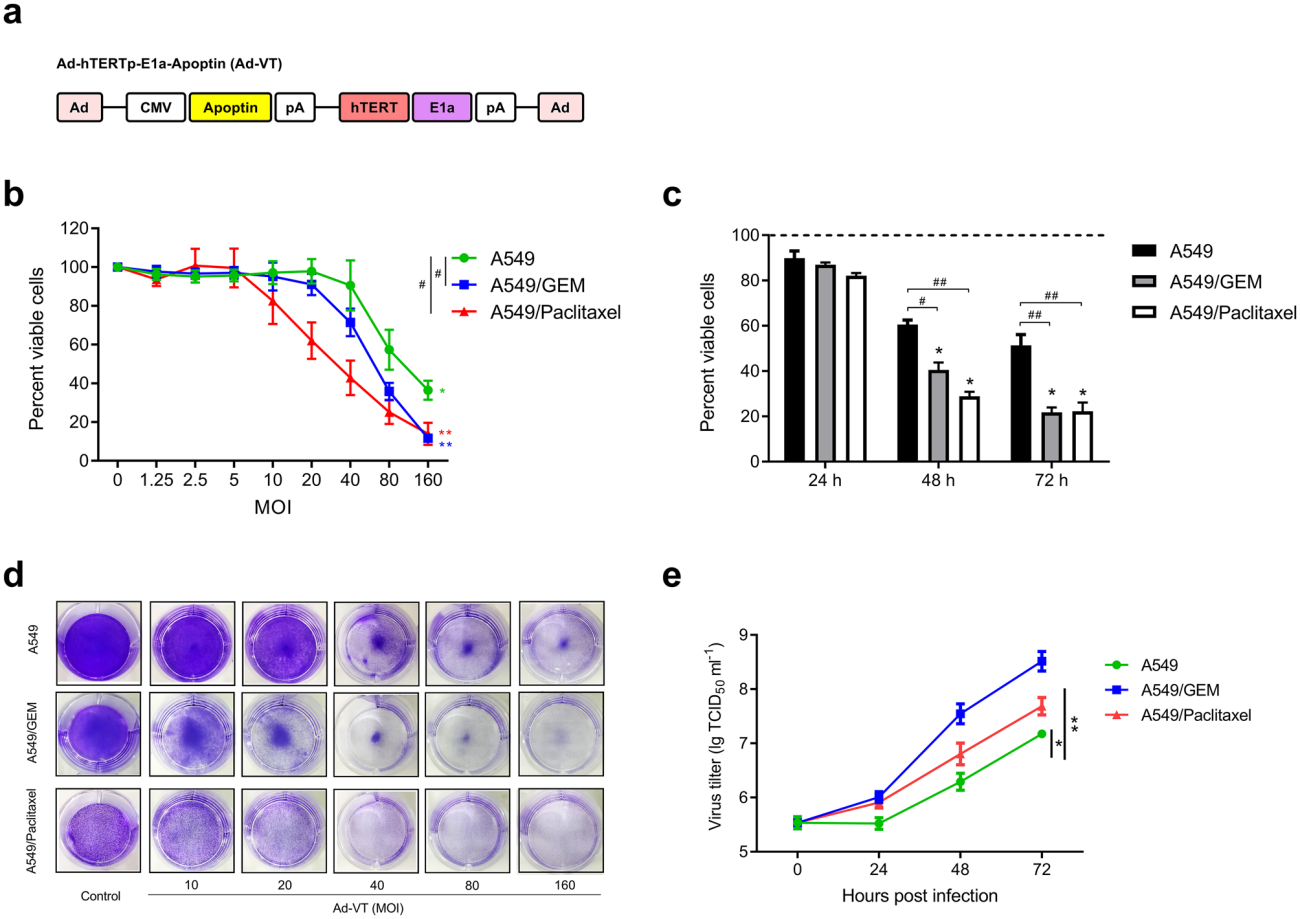
Comparative efficacy of Ad-VT on the inhibition of lung cancer cell growth and cell viability. **a** Schematic diagram showing the hTERTp-driven E1a cassette and the CMV-driven-apoptin expressing cassettes of Ad-hTERTp-E1a-Apoptin (Ad-VT). **b–c** Viability of A549, A549/GEM and A549/Paclitaxel cells as measured using the CCK-8 assay. **d** Crystal violet staining of cells treated with Ad-VT alone at several MOIs (twofold serial dilutions from 160 to 0.125 MOI). **e** Viral replication in A549, A549/GEM and A549/Paclitaxel lung cancer cells infected with Ad-VT at an MOI of 80 for 24 h, 48 h and 72 h. The data are presented as the means ± SD, n = 3. *p < 0.05, **p < 0.01 compared with the control group. ^#^*p* < 0.05, ^##^*p* < 0.01 compared with the corresponding A549 group

Ad-hTERTp-E1a-Apoptin (Ad-VT) contains an hTERTp-driven E1a cassette and a CMV-driven Apoptin (VP3 protein) cassette (Fig. 2a). To study the antitumor activity of Ad-VT, A549, A549/GEM and A549/Paclitaxel cells were infected with Ad-VT at different MOIs (twofold dilution from 160 MOI to 0.125 MOI) for 48 h. Pearson correlation analysis of CCK-8 results showed that the relative survival of various cells was related to the dose of Ad-VT (Fig. 2b). After infection with Ad-VT (MOI of 80) for 24, 48 and 72 h, the relative survival rates of the three cell lines were significantly decreased in a time-dependent manner (Fig. 2c). The three cell lines were then infected with different concentrations of Ad-VT (0, 10, 20, 30, 40, 80 and 160 MOI) for 48 h. The results showed that compared with A549 cells, Ad-VT cells had obvious cytotoxicity in A549/GEM and A549/Paclitaxel cells (Fig. 2d). In conclusion, the CCK-8 and crystal violet staining results showed that Ad-VT (MOI of 80) had an obvious killing effect on A549, A549/GEM and A549/Paclitaxel cells after 48 h of infection, and the killing effect on chemoresistant cells was significantly higher than that on A549 cells. Based on this finding, we used Ad-VT at an MOI of 80 in subsequent experiments. In addition, we observed the replication ability of Ad-VT in cells at different time points. The results showed that the titer of Ad-VT increased in a time-dependent manner in A549, A549/GEM and A549/Paclitaxel cells but that the replication level of Ad-VT in chemoresistant cells was higher than that in A549 cells at the same time points (Fig. 2e).

### Ad-VT significantly induced apoptosis in drug-resistant cell lines

**Fig. 3:**
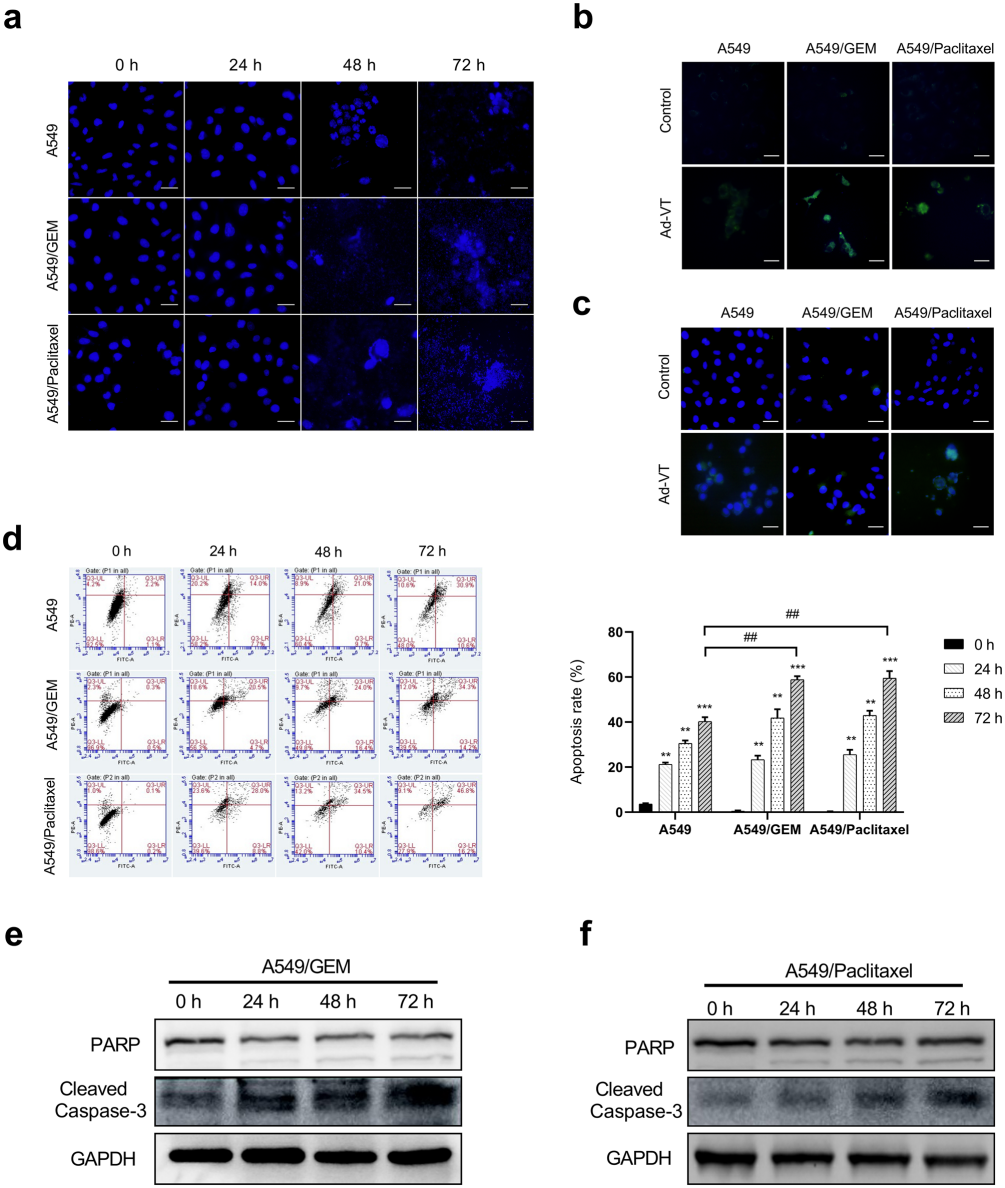
Ad-VT treatment induces apoptosis in lung cells. **a** Cells were treated with Ad-VT at an MOI of 80. After 24, 48 and 72 h, apoptotic cells were detected by Hoechst 33342 staining and observed under a fluorescence microscope. **b–c** Cells were treated with Ad-VT for 48 h, and apoptotic cells were detected by Annexin V-FITC staining (**b**) and TUNEL staining (**c**) under a fluorescence microscope. **d** Cells were incubated in the presence or absence of Ad-VT at an MOI of 80 for 0, 24, 48, and 72 h, and apoptosis was determined by flow cytometry with Annexin V-FITC/PI staining. **e–f** The molecular changes related to apoptosis in A549/GEM and A549/Paclitaxel cells were detected by Western blot with anti-PARP and anti-cleaved-caspase 3 antibodies. The values represent the means ± SD (n = 3). *p < 0.05, **p < 0.01, ***p < 0.001 compared with controls; ^##^*p* < 0.01 compared with the corresponding group. Magnification, 200 × . Scale bars: 50 µm

A549, A549/GEM and A549/Paclitaxel cell lines were treated with Ad-VT (MOI of 80), and apoptotic chromatin condensation was examined with Hoechst 33258 staining (Fig. 3a). Ad-VT-induced apoptotic chromatin condensation was obvious in A549/GEM and A549/Paclitaxel cells but minimal in A549 cells. We also assessed the apoptotic effects of Ad-VT through Annexin V-FITC and TUNEL staining (Fig. 3b, c), the results of which were consistent with those of Hoechst 33258 staining. These data indicate that apoptosis was the major mechanism of cell death induced by Ad-VT treatment, which was time dependent.

Quadrant statistical analysis showed that the number of apoptotic chemoresistant cells was significantly higher than the number of apoptotic A549 cells (Fig. 3d). In addition, we noted that Ad-VT significantly increased the expression of PARP and cleaved caspase-3 (Fig. 3e, f). These results indicated that Ad-VT induced caspase family-dependent apoptosis in A549/Paclitaxel and A549/GEM cells. Taken together with previous experiments, our data indicate that Ad-VT induced more significant apoptosis in A549/GEM and A549/Paclitaxel cells than in A549 cells.

### Elevated levels of autophagy in chemoresistant cell lines enhanced the effect of Ad-VT

**Fig. 4:**
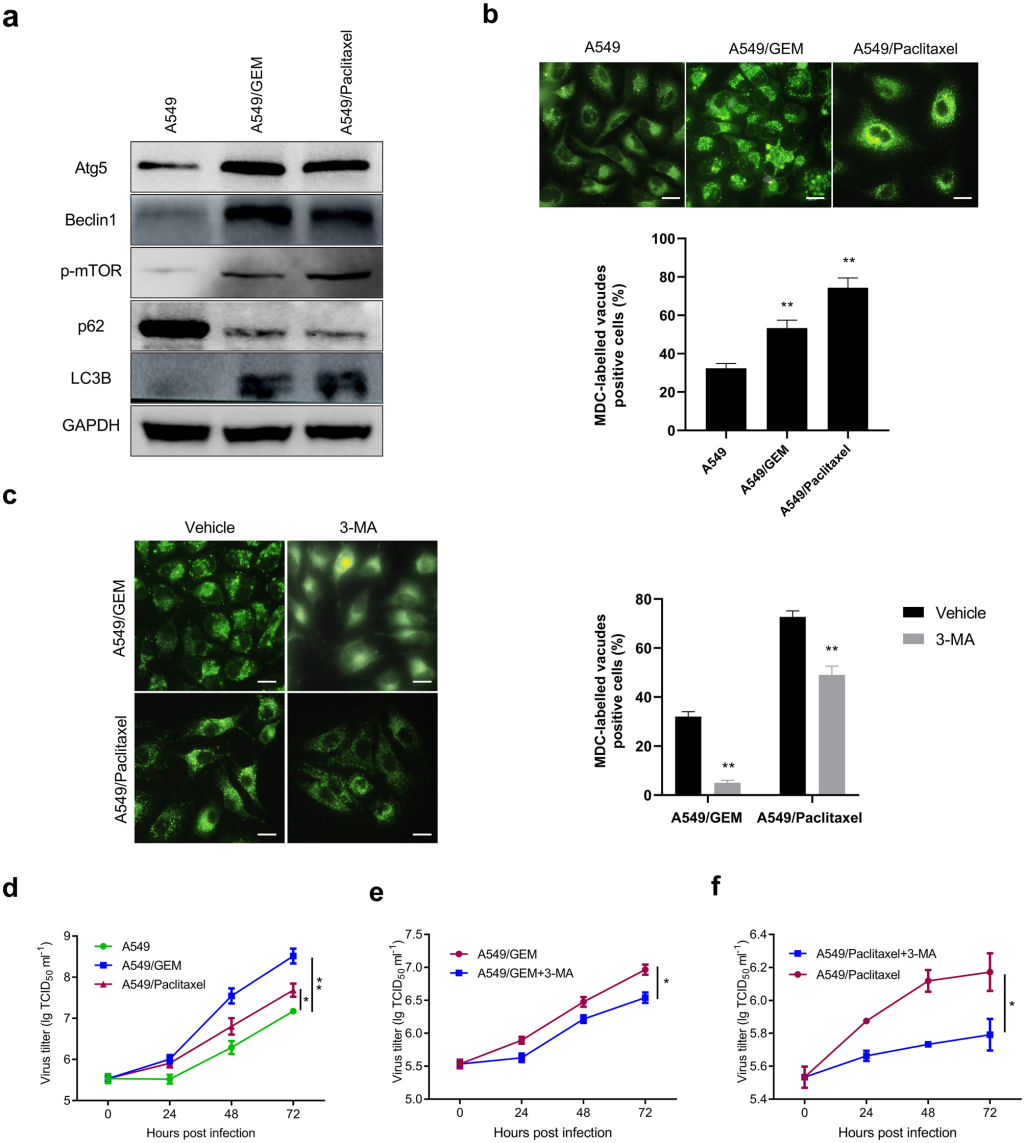
High levels of basal autophagy in drug-resistant cell lines enhanced the antitumor effect of Ad-VT. **a** The expression of the autophagy-related proteins ATG5, Beclin1, p-mTOR, p62 and LC3-II in lung cancer cells was evaluated by Western blotting. **b** Autophagic vacuoles in lung cancer cells were identified by MDC staining. Scale bars: 25 µm. **c** After A549/GEM and A549/Paclitaxel cells were treated with 3-MA, autophagic vacuoles were detected by MDC staining. **d** Detection of viral replication in A549, A549/GEM and A549/Paclitaxel lung cancer cells infected with Ad-VT at different time points. **e–f** After 3-MA treatment, viral replication in A549/GEM and A549/Paclitaxel cells was detected at different time points after Ad-VT (MOI of 80) infection, n = 8 for each group. The data are presented as the means ± SD, *p < 0.05, **p < 0.01 compared with the controls

Autophagy is reported to play an important role in the drug resistance of cancer cells, including NSCLC cells [16]. To detect whether drug-resistant lung cancer cells have high levels of autophagy, Western blot and fluorescence microscopy were performed. The results showed that the expression levels of the autophagy-related proteins ATG5, Beclin1, p-mTOR and LC3B in A549/GEM cells were significantly higher than those in A549 cells (Fig. 4a), and the expression trend in A549/Paclitaxel cells was similar to that in A549/GEM cells. The expression of p62 protein in A549/GEM and A549/Paclitaxel cells was significantly lower than that in A549 cells (Fig. 4a). MDC, a known tracer for autophagic vesicles and that emits green fluorescence, is taken up by cells and selectively binds to autophagic vacuoles [17]. The MDC staining results in Fig. 4b show that punctate MDC-positive fluorescent particles were present in the cytoplasm of A549/GEM or A549/Paclitaxel cells, indicating that the drug-resistant cell lines were more positive for autophagosomes than were A549 cells. When 3-MA, an autophagy inhibitor, was added to A549/GEM and A549/Paclitaxel cells, the number of autophagosome-positive cells was significantly reduced compared with that of A549 cells. These results indicate that chemoresistant lung cancer cells have higher autophagy levels than their parental cells. Finally, we investigated whether autophagy could affect virus replication and, consequently, the effect of OVs in chemoresistant cells. After 48 h of treatment with 3-MA, the Ad-VT viral titer in chemoresistant cell lines decreased significantly, suggesting that autophagy significantly increased the level of viral replication in A549/GEM and A549/Paclitaxel cells (Fig. 4d, e and f).

### Ad-VT combined with gemcitabine or paclitaxel inhibited the growth and reduced the drug resistance of A549/GEM or A549/Paclitaxel cells

**Fig. 5:**
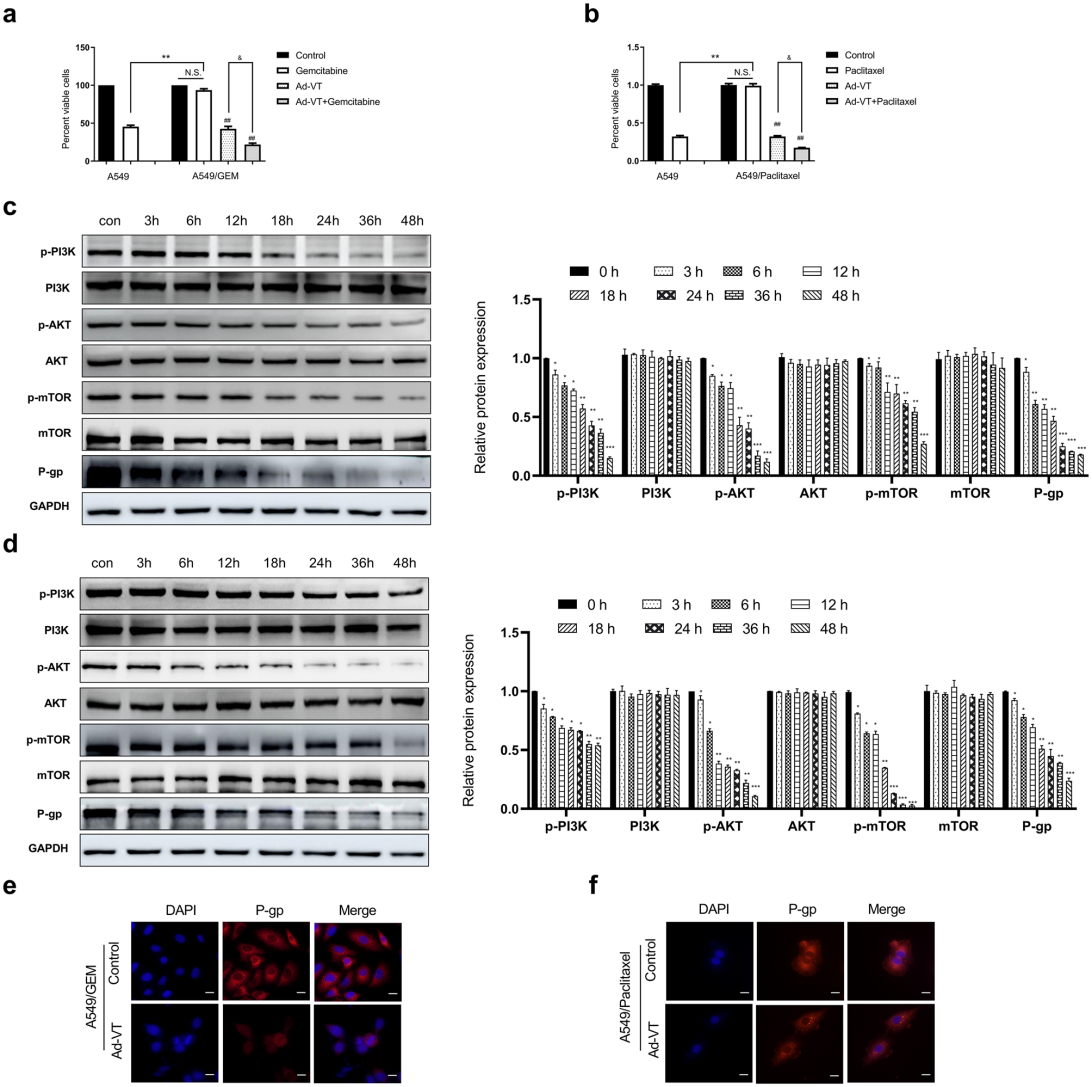
Ad-VT combined with gemcitabine or paclitaxel can inhibit the growth of A549/GEM and A549/Paclitaxel cells and reduce their drug resistance. **a–b** A549 cells were treated with Ad-VT (MOI of 80); A549/GEM cells were treated with gemcitabine (10 µg/ml) and Ad-VT (MOI of 80) either alone or in combination; and A549/Paclitaxel cells were treated with paclitaxel (10 µg/ml) and Ad-VT (MOI of 80) either alone or in combination. All treatments lasted 48 h, and viability was examined by the CCK-8 assay. **c–d** Western blotting was used to analyze the expression levels of p-PI3k, PI3K, p-mTOR, mTOR, p-Akt, Akt and P-gp in A549/GEM and A549/Paclitaxel cells treated with Ad-VT (MOI of 80) at different time points. All data are shown as the means ± SD, n = 3. **e–f** Immunofluorescence analysis of P-gp protein expression (red fluorescence) and the nucleus (blue fluorescence, DAPI staining) in A549/GEM and A549/Paclitaxel cells treated with Ad-VT (MOI of 80) for 24 h. Scale bars: 25 µm. In untreated control cells, cell viability was considered to be 100%. The data are presented as the means ± SD, *p < 0.05, **p < 0.01 compared with controls. ^#^*p* < 0.05, ^##^*p* < 0.01 compared with the corresponding group. ^&^*p* < 0.05 compared with the indicated group

The effects of Ad-VT and gemcitabine on A549 or A549/GEM cells were compared using CCK-8 assay. The results showed that gemcitabine alone could significantly reduce the survival rate of A549 cells but had no significant effect on A549/GEM cells, which supported the resistance of A549/GEM cells to gemcitabine. When gemcitabine was combined with Ad-VT, the survival of A549/GEM cells was significantly lower than that in the gemcitabine alone and control groups (Fig. 5a). In addition, we studied the effect of Ad-VT combined with paclitaxel on A549/Paclitaxel cells. Paclitaxel significantly decreased the survival of A549 cells but had no significant effect on A549/Paclitaxel cells. However, the combination of Ad-VT and paclitaxel significantly reduced the survival of A549/Paclitaxel cells compared with paclitaxel alone and control treatment (Fig. 5b).

Activation of Akt, a downstream constituent of the PI3K pathway, can be observed in many cancer cells and tissues. There is evidence that the PI3K/Akt pathway is related to the drug resistance of NSCLC, which affects the sensitivity of lung cancer cells to cisplatin [18]. It has also been reported that downregulation of P-gp expression via inhibition of the PI3K/Akt/mTOR signaling pathway can induce apoptosis in multidrug-resistant cells [19, 20]. According to our previous work, Ad-VT regulates P-gp protein levels by affecting the PI3K/Akt/mTOR signaling pathway. We used Ad-VT (MOI of 80) to infect cells and then detected the expression of related proteins at different times after infection (0, 3, 6, 12, 18, 24, 36 and 48 h) (Fig. 5c, d). The Western blotting results showed that Ad-VT significantly inhibited p-PI3K, p-Akt, p-mTOR and P-gp levels in a time-dependent manner. The immunofluorescence results revealed that Ad-VT induced the downregulation of P-gp protein expression in A549/GEM and A549/Paclitaxel cells (Fig. 5e, f) consistent with the Western blot results. In conclusion, Ad-VT downregulates P-gp by reducing the expression of key proteins in the PI3K/Akt/mTOR signaling pathway, thereby interfering with the resistance of A549/GEM cells to gemcitabine or A549/Paclitaxel cells to paclitaxel.

### Ad-VT inhibited tumor growth in vivo

**Fig. 6:**
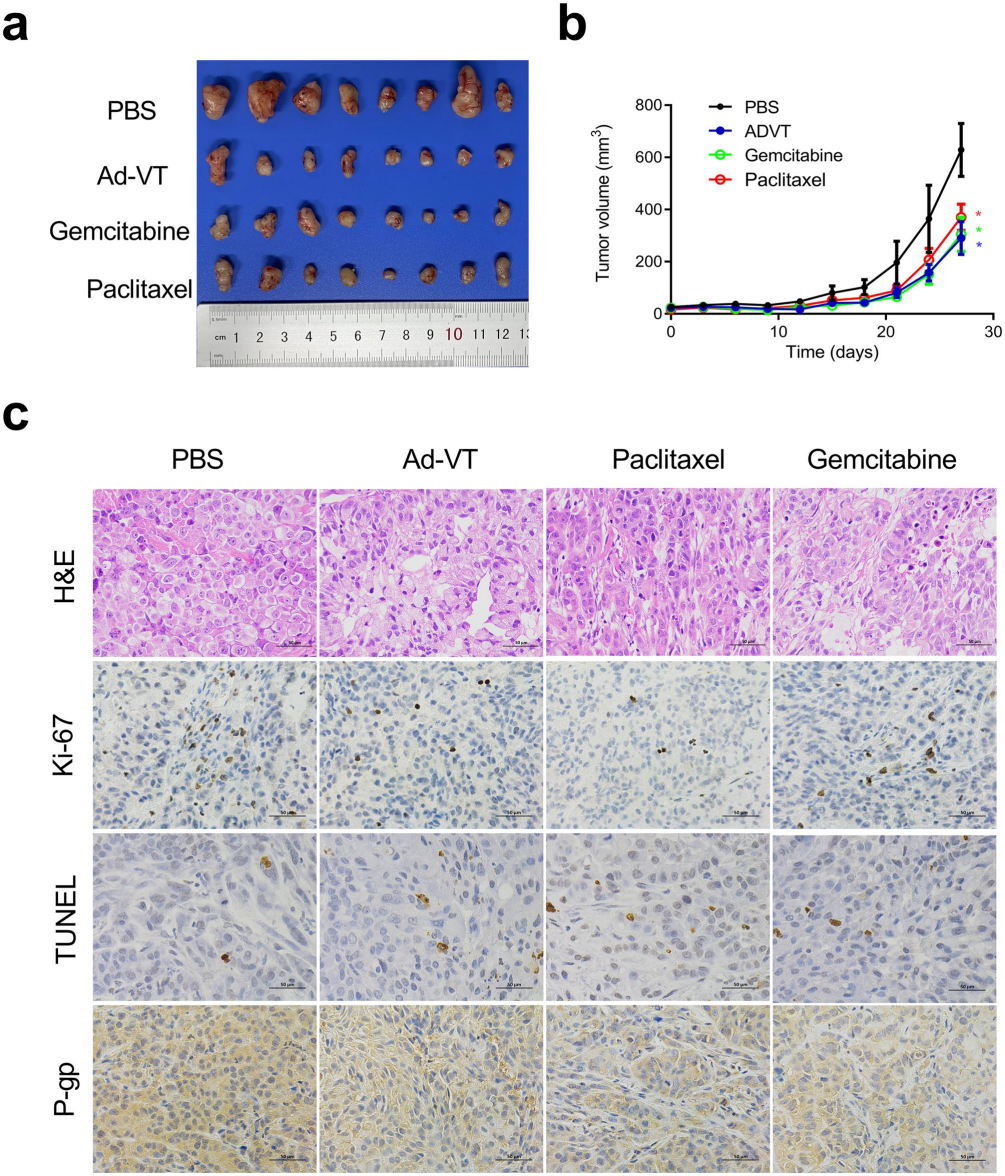
Effect of Ad-VT, gemcitabine and paclitaxel on A549 cell-derived xenografts in a BALB/c nude mouse model. **a** Xenograft tumors from nude mice treated with Ad-VT, gemcitabine and paclitaxel were excised, imaged and measured. **b** The tumor volume curves of mice treated with gemcitabine (10 mg/kg), paclitaxel (10 mg/kg), Ad-VT (1 × 10^9^ plaque-forming units) and PBS, n = 10/group, *p < 0.05. **c** The histological features of sections from A549 cell-derived tumors were characterized by H&E staining. Ki-67 and P-gp expression in the xenografts was detected by immunohistochemical staining, and TUNEL staining was performed. Images are typical of 3 independent assays. Magnification, 400 × . Scale bars: 50 µm

To further study the antitumor effects of Ad-VT, gemcitabine and paclitaxel, a xenograft model was established in nude mice. Compared with the control treatment, Ad-VT, gemcitabine and paclitaxel treatment resulted in a significant decrease in tumor volume (Fig. 6a, b), suggesting that Ad-VT, gemcitabine and paclitaxel have inhibitory effects on lung cancer. IHC showed that compared with the PBS control group, the Ad-VT, gemcitabine and paclitaxel treatment groups had decreased Ki-67 expression in lung cancer tissues and increased TUNEL staining (Fig. 6c). By IHC, we also found that the protein expression of P-gp in the Ad-VT, gemcitabine or paclitaxel groups was lower than that in the PBS group (Fig. 6c). These results indicate that Ad-VT, gemcitabine and paclitaxel can inhibit the proliferation of tumor cells, promote apoptosis, and inhibit P-gp expression in vivo.

### Ad-VT increased the sensitivity of chemoresistant xenografts to gemcitabine and paclitaxel in a BALB/c nude mouse model

**Fig. 7:**
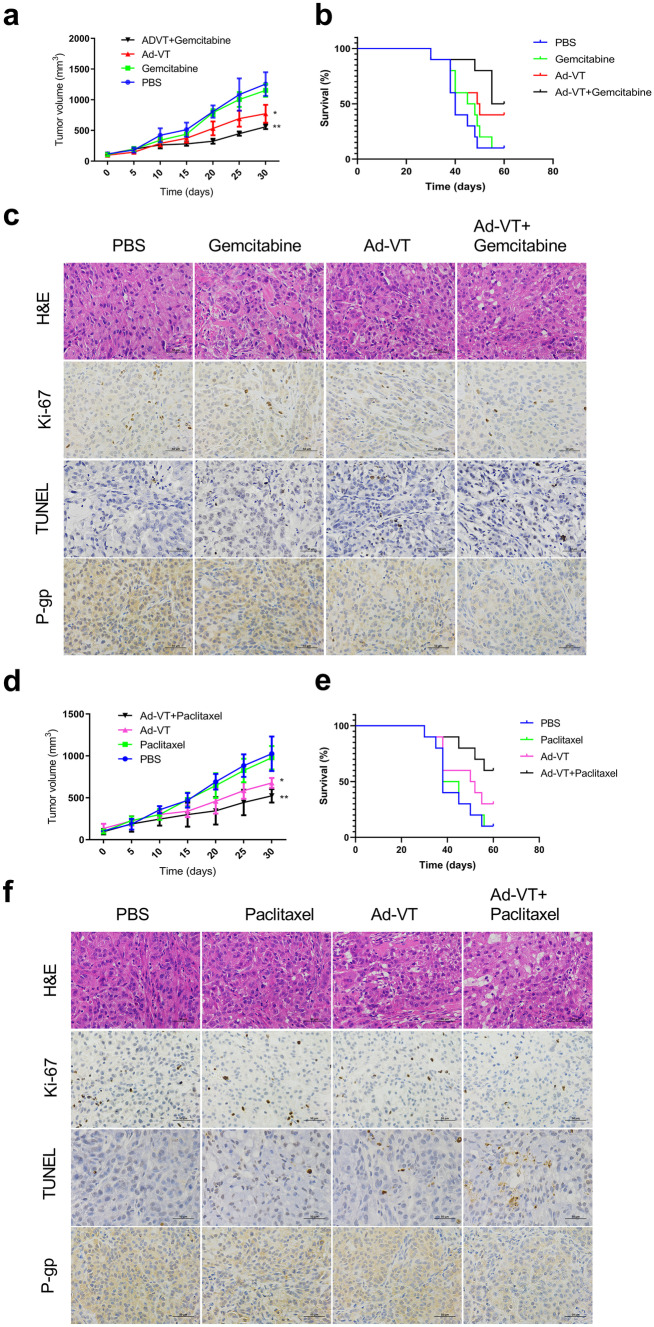
The synergistic effect of Ad-VT with gemcitabine or paclitaxel on A549/GEM- and A549/Paclitaxel-derived xenografts in a BALB/c nude mouse model. **a**,** d** Growth curve of tumor volumes in the different treatment groups. Each data point indicates the mean ± SD. n = 7, *p < 0.05, **p < 0.01 vs. the PBS group. **b**,** e** The survival rate of mice in the different treatment groups. **c**,** f** The histological features of subcutaneous tumors derived from A549/GEM and A549/Paclitaxel cells were characterized by H&E staining. Ki-67 and P-gp expression in the xenografts was detected by immunohistochemical staining, and TUNEL staining was performed. Images are typical of 3 independent assays. Magnification, 400 × . Scale bars: 50 µm

To further study the therapeutic effect of Ad-VT combined with gemcitabine or paclitaxel, we established a xenograft tumor model in nude mice. Figure 7a illustrates the effects of the combination of Ad-VT and gemcitabine on the growth of A549/GEM-derived tumors. The results indicate that compared with the control group, the gemcitabine-treatment group had only slightly smaller tumors, indicating that gemcitabine has a less pronounced effect on drug-resistant tumors. The tumor volume of the Ad-VT group was significantly smaller than that of the control group, and there a significant difference on day 30; these data indicated that Ad-VT was a significant inhibitory effect on the growth of drug-resistant tumors (*p* < 0.05). As expected, the tumor volume of the Ad-VT plus gemcitabine group grew more slowly and was significantly smaller than that of the control, gemcitabine and Ad-VT groups, demonstrating that the combination therapy had a significant inhibitory effect on tumor growth (*p* < 0.05). After combined treatment, the survival time of mice was significantly prolonged. The combination of Ad-VT and gemcitabine improved the survival rate and prolonged the survival time of mice (Fig. 7b).

The synergistic effect of Ad-VT and gemcitabine was further confirmed by histological assessments of tumors in each group (Fig. 7c). The H&E staining results showed that in the Ad-VT combined with gemcitabine group, there was nucleolysis, pyknosis and an increased number of cytoplasmic vacuoles, and the inhibition of tumor cell proliferation was the most obvious. The protein expression of P-gp in the Ad-VT combined with gemcitabine group was significantly lower than that in either monotherapy group or the control group (*p* < 0.01). The TUNEL results revealed that Ad-VT combined with gemcitabine treatment was significantly more effective as an antitumor therapy than either monotherapy group or the control group. Assessment of Ki-67 expression showed that Ad-VT combined with gemcitabine treatment resulted in significantly lower Ki-67 expression than did either monotherapy and control treatment. These results consistently indicate that Ad-VT combined with gemcitabine has the strongest antitumor activity against A549/GEM xenografts in nude mice compared with that of the control treatment.

Next, we further conducted the same experiments described above with Ad-VT combined with paclitaxel in A549/Paclitaxel cell-derived xenografts. As shown in Fig. 7d, e and f, as expected, the tumor volume of the Ad-VT combined with paclitaxel group on days 20, 25, and 30 was smaller than that of either monotherapy group or the control group, with a statistically significant difference observed on day 56. The survival rate at day 56 was 10% in the control group, 10% in the paclitaxel group, 30% in the Ad-VT group, and 60% in the combination treatment group. These results showed that the survival rate of tumor-bearing mice in the combined treatment group was significantly higher than that in the monotherapy groups and the control group. The H&E results showed that nucleolysis and the number of cytoplasmic vacuoles were increased in the combination treatment group (Fig. 7f). The TUNEL results indicated that Ad-VT combined with paclitaxel was significantly stronger than the monotherapy groups and the control group in inducing cell death. The Ki-67 results revealed that tumor proliferation in the Ad-VT combined with paclitaxel group was significantly lower than that in the Ad-VT alone and control groups. Moreover, Ad-VT combined with paclitaxel significantly reduced the protein expression of P-gp. Compared with the control treatment, Ad-VT combined with paclitaxel had the most effective antitumor activity in the A549/Paclitaxel xenograft tumor model in nude mice.

## Discussion

Chemoresistance has always been a major obstacle to the success of tumor chemotherapy. Although gemcitabine and paclitaxel, as first-line antitumor drugs, have been widely used in the treatment of NSCLC, chemotherapy failure is often caused by the development of drug resistance. To overcome MDR, a large number of studies have been carried out to elucidate the complex mechanism(s) involved. The high expression of multidrug resistance gene 1 (MDR1) in tumor tissue has been reported to be related to poor prognosis and MDR in tumor patients [21].

P-gp mediates MDR, and P-gp overexpression in drug-resistant tumor cells is one of the driving factors of tumor resistance [22]. High and persistent expression of P-gp increases the resistance of the lung squamous cell carcinoma cell line CH27 to doxorubicin [22]. At present, many drugs have been developed and applied to prevent or mitigate chemotherapy resistance in lung cancer, including calcium channel blockers, antiarrhythmic drugs and some components of traditional Chinese medicine [23]. Although there are drugs that can temporarily increase the concentration of chemotherapeutic drugs to sufficient levels that will kill tumor cells, they cannot be widely used in the clinic due to their instability, nonspecificity and adverse effects. Therefore, finding effective drugs or treatment strategies to inhibit or reverse MDR is the key to improving the survival of lung cancer patients.

With advances in molecular biology and gene engineering technologies, many novel treatment schemes have been developed, including gene targeting and OV therapy. Compared with other cancer treatment methods, OVs have a broad antitumor spectrum, are safe and reliable, are less toxic with fewer adverse effects, and exert synergistic effects with other anticancer drugs [24]. Therefore, the development and clinical application of adenoviruses as drug vectors have been widely studied, and progress has been made. Adenovirus-based tumor-targeted therapy is predicted to replace traditional tumor therapy. In the present study, we investigated the effects of Ad-VT (Ad-hTERTp-E1a-Apoptin), a new recombinant OV that can specifically replicate in tumor cells and express apoptin protein efficiently (thus playing an effective role in tumor cell death) [25], on gemcitabine-resistant and paclitaxel-resistant lung cancer cells in vitro and on their corresponding xenograft tumor models in vivo. Ad-VT was observed to enhance the chemosensitivity of drug-resistant cells in vitro and in vivo. Our previous studies have shown that Ad-VT can inhibit a variety of tumor cells and has good safety [26]. The tumor-specific promoter hTERTp can activate the replication and/or expression of certain genes in tumor cells and is located upstream of the adenovirus promoter E1a gene and apoptin gene in Ad-VT. This recombinant design bestows Ad-VT with specific replication and killing abilities.

In the present study, we found that Ad-VT has a high replication rate in drug-resistant lung cancer cells and can not only induce apoptosis of drug-resistant lung cancer cells but also reduce the expression of the P-gp protein. It is speculated that Ad-VT is a promising drug for the treatment of gemcitabine- or paclitaxel-resistant NSCLC, with previous studies reporting that persistent elevated P-gp expression increases the resistance of DOX-resistant CH27 cells to DOX [27]. Our Western blot results showed that P-gp expression in A549/GEM and A549/Paclitaxel cells was higher than that in parental A549 cells (Fig. 1a), which was consistent with the drug resistance characteristics of A549/GEM and A549/Paclitaxel cells.

The Kaplan–Meier Plotter database showed that P-gp expression in lung cancer tissues was significantly higher than that in normal lung tissues (Fig. 1b). It is generally believed that the ability to induce apoptosis is one of the most basic elements in the development of antitumor drugs [28], and apoptosis activation is related to the cytotoxic effect of chemotherapy on tumor cells. In this study, our results show that Ad-VT not only inhibits proliferation but also induces apoptosis in A549, A549/GEM and A549/Paclitaxel cells in a dose- and time-dependent manner; furthermore, the rates of proliferation inhibition and apoptosis induction were higher in A549/GEM and A549/Paclitaxel cells than in A549 cells.

An increasing number of studies have indicated that autophagy plays an important role in tumor drug resistance. Autophagy is an evolutionarily conserved process that provides metabolic support for the degradation and renewal of long-lived proteins and dysfunctional organelles to maintain cell homeostasis. Autophagy occurs continuously at basal metabolic levels in normal cells and is upregulated in response to stressors such as starvation, oxidative stress or cytotoxic drug therapy [29]. It has also been reported that tumor cells can induce chemotherapy resistance via autophagy [30–32]. LC3-II is a reliable marker for monitoring autophagy and autophagy-related processes in mammals [33, 34], and increased levels of p62 (sqstm1) indicate that autophagy is blocked. When LC3-II is upregulated, p62 is downregulated, indicating that autophagy is active, whereas decreased LC3-II and increased p62 levels indicate that autophagy is inhibited [28]. mTOR is an evolutionarily conserved serine/threonine kinase that regulates autophagy [35]. ATG5 accumulation leads to negative feedback to signals upstream of mTOR, resulting in reductions in the level of phosphorylated mTOR and consequent activation of the autophagic pathway [36]. Our results showed that compared with A549 cells, A549/GEM and A549/Paclitaxel cells had increased expression levels of Atg5, p-mTOR and LC3-II and decreased expression of p62 (Fig. 4a). These data are in agreement with the literature stating that autophagy is enhanced in drug-resistant cell lines. In addition, MDC-labeled vacuole analysis revealed that A549/GEM and A549/Paclitaxel cells had stronger basal autophagy activity than parental A549 cells. This result indicated that resistance to gemcitabine and paclitaxel was related to enhanced autophagy that is, autophagy is a survival mechanism of gemcitabine- or paclitaxel-resistant cell lines against chemotherapy drugs. Research shows that the autophagy inducer rapamycin improves the oncolytic efficacy of Newcastle disease OVs in drug-resistant lung cancer cells through enhanced autophagy [33]. The results of the virus titer assay showed that Ad-VT had a stronger replication ability in A549/GEM and A549/Paclitaxel cell lines than in A549 cells (Fig. 4d). The autophagy inhibitor 3-MA decreased the level of autophagy in a drug-resistant lung cancer cell line and weakened the replication ability of Ad-VT, thus affecting the cytotoxicity of Ad-VT (Fig. 4d, e and f). Combined with the above results, these data suggest that autophagy contributes to the oncolytic effect of Ad-VT in drug-resistant lung cancer cell lines.

Increasingly, studies have shown that the PI3K/Akt/mTOR signaling pathway is related to tumor drug resistance [37–39]. Akt inhibitors increase the sensitivity of cancer cells to chemotherapy and radiotherapy [40, 41], and inhibitors of PI3K and mTOR play an important role in the treatment and drug resistance of cancer [41]. Some studies have reported that resveratrol can downregulate P-gp expression by inhibiting the PI3K/Akt/mTOR signaling pathway and subsequently reduce the drug resistance of cancer cells [19, 20], suggesting that the PI3K/Akt/mTOR signaling pathway is closely related to drug resistance. This conclusion is logical, as P-gp (a transmembrane drug transporter) is involved in drug resistance and is regulated by the PI3K/Akt/mTOR signaling pathway. Our results showed that Ad-VT downregulated the expression levels of not only p-pI3k, p-Akt and p-mTOR pathway proteins but also P-gp protein in chemoresistant A549/GEM and A549/Paclitaxel cancer cell lines (Fig. 5c), resulting in decreased PI3K/Akt/mTOR signaling pathway function and reduced chemotherapy resistance of drug-resistant lung cancer cell lines. These data suggest that Ad-VT plays a role in chemotherapy resistance at the molecular level by affecting the protein expression of constituents of the PI3K/Akt/mTOR signaling pathway, which can be exploited to improve the sensitivity of drug-resistant lung cancer cell lines to chemotherapy.

The nude mouse xenograft tumor model was established with A549, A549/GEM and A549/Paclitaxel cells. We observed that in xenografts derived from A549 cells, the tumor volumes of the Ad-VT, gemcitabine, and paclitaxel groups were significantly reduced compared with those of the control group (Fig. 6a, b), with IHC showing that Ki-67 and P-gp expression was decreased and TUNEL staining was increased (Fig. 6c). In xenografts derived from A549/GEM cells, Ad-VT combined with gemcitabine caused significant tumor growth inhibition, which was significantly stronger than that caused by either gemcitabine or Ad-VT alone and by the control treatment. Moreover, compared with those from the monotherapy and control groups, the xenografts from the Ad-VT and combination gemcitabine group exhibited significant nucleolysis, increased number of vacuoles, decreased Ki-67 expression, increased TUNEL staining, and decreased P-gp expression. In xenografts derived from A549/Paclitaxel cells, the results were similar to those of A549/GEM xenograft tumors. In conclusion, Ad-VT alone or in combination with gemcitabine or paclitaxel had obvious antitumor effects in a chemoresistant tumor model. More importantly, Ad-VT can improve the chemosensitivity of chemoresistant lung cancer cells in vivo. The inhibitory effect of gemcitabine and paclitaxel on A549/GEM and A549/Paclitaxel cells, respectively, was significantly enhanced when combined with Ad-VT, indicating that these combinations exhibit a synergistic anticancer effect, suppress the drug resistance to chemotherapy drugs, and reduce the side effects of gemcitabine and paclitaxel. Therefore, in future research and clinical applications, the effectiveness, safety and optimization of the administration of chemotherapeutic drugs in combination with Ad-VT shoud be the main goals to better develop and utilize the application of Ad-VT in drug-resistant tumors.

## Conclusions

Ad-VT can significantly inhibit the proliferation of gemcitabine- and paclitaxel-resistant lung cancer cells mainly by inducing apoptosis. Ad-VT can also improve the sensitivity of drug-resistant lung cancer cells to chemotherapy drugs. This mechanism can be divided into two aspects: first, the replication of Ad-VT increases because of the strong autophagy activity of drug-resistant cells; and second, Ad-VT mainly reduces the chemotherapy resistance of drug-resistant lung cancer cells by inhibiting PI3K/Akt/mTOR pathway function and downregulating P-gp expression. Therefore, our study suggests that the combination of Ad-VT and chemotherapeutic drugs is a promising therapeutic strategy to overcome chemoresistance.

**Table 1:** The resistance index (RI) of various compounds in A549/GEM and A549/Paclitaxel cells (mean ± SD, n=3)

Compounds	IC 50 (nm)			RI
	A549	A549/GEM	A549/Paclitaxel	
Paclitaxel	31.44±1.72	20.22±1.480	3193±207.6	A549/GEM:1 A549/Paclitaxel:103
Gemcitabine	481.3±88.35	8369±474.6	406.2±87.11	A549/GEM:17 A549/Paclitaxel:1

## Data Availability

The datasets generated and/or analyzed during the current study are available from the corresponding author on reasonable request.
